# Sustained Vascular Inflammatory Effects of SARS-CoV-2 Spike Protein on Human Endothelial Cells

**DOI:** 10.1007/s10753-024-02208-x

**Published:** 2024-12-31

**Authors:** Mitra Gultom, Lin Lin, Camilla Blunk Brandt, Anastasia Milusev, Alain Despont, Jane Shaw, Yvonne Döring, Yonglun Luo, Robert Rieben

**Affiliations:** 1 Department for Biomedical Research, University of Bern, Bern, Switzerland; 2 Department of Biomedicine, Aarhus University, Aarhus, Denmark; 3 Steno Diabetes Center Aarhus, Aarhus University Hospital, Aarhus, Denmark; 4 Department of Angiology, Inselspital, Bern University Hospital, Bern, Switzerland; 5 Institute for Cardiovascular Prevention (IPEK), Ludwig Maximilian University, Munich, Germany; 6 German Centre for Cardiovascular Research (Deutsches Zentrum Für Herz-Kreislauf-Forschung, DZHK), Munich Heart Alliance Partner Site, Munich, Germany

**Keywords:** SARS-CoV-2, Spike protein, Endothelial cells, Vascular inflammation

## Abstract

Severe acute respiratory syndrome coronavirus 2 (SARS-CoV-2) infection has been associated with systemic inflammation and vascular injury, which contribute to the development of acute respiratory syndrome (ARDS) and the mortality of COVID-19 infection. Moreover, multiorgan complications due to persistent endothelial dysfunction have been suspected as the cause of post-acute sequelae of SARS-CoV-2 infection. Therefore, elucidation of the vascular inflammatory effect of SARS-CoV-2 will increase our understanding of how endothelial cells (ECs) contribute to the short- and long-term consequences of SARS-CoV-2 infection. Here, we investigated the interaction of SARS-CoV-2 spike protein with human ECs from aortic (HAoEC) and pulmonary microvascular (HPMC) origins, cultured under physiological flow conditions. We showed that the SARS-CoV-2 spike protein triggers prolonged expression of cell adhesion markers in both ECs, similar to the effect of TNF-α. SARS-CoV-2 spike treatment also led to the release of various cytokines and chemokines observed in severe COVID-19 patients. Moreover, increased binding of leucocytes to the endothelial surface and a procoagulant state of the endothelium were observed. Transcriptomic profiles of SARS-CoV-2 spike-activated HPMC and HAoEC showed prolonged upregulation of genes and pathways associated with responses to virus, cytokine-mediated signaling, pattern recognition, as well as complement and coagulation pathways. Our findings support experimental and clinical observations of the vascular consequences of SARS-CoV-2 infection and highlight the importance of EC protection as one of the strategies to mitigate the severe effects as well as the possible post-acute complications of COVID-19 disease.

**Supplementary Information:**

The online version contains supplementary material available at 10.1007/s10753-024-02208-x.

## Introduction

Although severe acute respiratory syndrome coronavirus 2 (SARS-CoV-2) primarily infects the respiratory tract, there is evidence that the causative agent of COVID-19 disease directly interacts with the vasculature [1–3]. The observed thrombotic symptoms in severe COVID-19 patients, with arterial and venous thrombosis contributing to their mortality, highlight the substantial consequences of SARS-CoV-2 on the endothelium [4–6]. Beyond the lungs, vascular coagulopathy and inflammation leading to cardiovascular and neurological complications have been reported in kidney, heart, and brain [7–10]. Moreover, preexisting cardiovascular disorders associated with endothelial dysfunction are one of the main comorbidities correlated with severe COVID-19 disease outcomes and deaths [11, 12].

Endothelial cells (ECs) are constantly exposed to plasma components and have a crucial role in maintaining vascular homeostasis, including regulating the coagulation state, as well as local and general inflammation. Although direct infection of SARS-CoV-2 of ECs has been described, findings regarding the susceptibility of ECs for infection and the expression of the receptor angiotensin-converting enzyme 2 (ACE2) and transmembrane protease serine 2 (TMPRSS2) are controversial [13–16]. Several reports described that SARS-CoV-2 particles, including the spike protein, can interact directly with the endothelium, leading to barrier damage and vascular dysfunction [17–19]. In the lungs, SARS-CoV-2 spike protein alone can induce infection-like injury and vascular inflammatory responses, including increases in endothelial adhesion molecule expression and recruitment of immune cells [20–22]. Circulating viral particles in the plasma of COVID-19 patients correlated with disease severity, indicating a high incidence of endothelial exposure, also beyond the respiratory tract [6, 23, 24]. Moreover, spike protein present in the plasma can be taken up and deposited in various organs, suggesting that systemic spread of SARS-CoV-2 is facilitated by ECs [25, 26].

Several studies have documented possible mechanisms through which SARS-CoV-2 causes vascular injury and endothelial dysfunction, including downregulation of ACE2 and disruption of mitochondrial function [6, 22]. Others have described that the vascular damage effect of SARS-CoV-2 spike protein is caused by endothelial glycocalyx disruption and engagement of integrins, leading to the activation of TGF-β signaling [18, 27, 28]. Robles et al. showed that SARS-CoV-2 spike protein can induce the nuclear translocation of NF-kB, leading to expression of procoagulant and proinflammatory responses of ECs [29].

The presence of biomarkers for systemic inflammation and an increased procoagulant state in the plasma are prominently observed in acute COVID-19 infection [1, 4, 30, 31]. Moreover, systemic inflammation was still observed when viral infection was cleared in COVID-19 patients, potentially contributing to the multi-organ disorders in the post-acute sequelae of SARS-CoV-2 (PASC), also known as “Long COVID” [32–35]. In this context, persistent endothelial dysfunction has been identified as a significant driver of prolonged non-respiratory symptoms and PASC [36, 37]. Therefore, understanding the extent of vascular inflammation as well as the sustained changes to the endothelial functions caused by SARS-CoV-2 is necessary to get a better grip on the consequences of COVID-19 infection.

In this study, we investigated the interaction of SARS-CoV-2 spike protein with primary human ECs from two anatomical origins: aortic (HAoEC) as well as lung microvascular (HPMC), which were cultured under physiological flow to simulate the vascular environment. We showed that SARS-CoV-2 spike protein elicited prolonged inflammatory responses in both the macro- and microvascular ECs. We also investigated pathways mediating the interaction of SARS-CoV-2 spike protein and cellular dynamic of ECs post-interaction, elucidating possible persistent effect of SARS-CoV-2 on the endothelium.

## Materials and Methods

### Primary Endothelial Cells and Cell Lines

Primary human pulmonary microvascular cells (HPMC, Promocell, C-12281) and primary human aortic endothelial cells (HAoEC, Promocell, C-12271) were obtained from a commercial supplier. For expansion, the cells were grown in a fibronectin-coated flask using complete media (Endothelial growth medium MV2 (Promocell, C-22011), supplemented with heat-inactivated fetal bovine serum (FBS) to a total concentration of 10%, 100 IU penicillin, and 100 μg/ml streptomycin). Cells were maintained at 37 °C in a humidified incubator supplied with 5% CO_2_. For each experiment, cells at passage 5 or 6 were used.

Vero-E6 cells (kindly provided by M. Müller, Charite Berlin) were cultured on Dulbecco’s modified Eagle’s medium-GlutaMAX (DMEM-GlutaMAX, Gibco) supplemented with 10% heat-inactivated FBS, 1 × non-essential amino acids (Gibco), 100 IU penicillin and 100 μg/ml streptomycin. Cells were maintained at 37 °C in a humidified incubator with 5% CO_2_.

Calu-3 cells (American Type Culture Collection (ATCC), HTB-55) were propagated on DMEM-GlutaMAX supplemented with 10% heat-inactivated FBS, 100 IU/mL penicillin, 100 μg/ml streptomycin, and 1 × non-essential amino acids. Cells were maintained at 37 °C in a humidified incubator with 5% CO_2_.

### SARS-CoV-2 Viral Stock

SARS-CoV-2 (SARS-CoV-2/München-1.1/2020/929) was propagated in Calu-3 cells. Briefly, 24 h before infection, Calu-3 cells were seeded in T75 flasks. The day after, they were infected with the virus stock at the multiplication of infection (MOI) of 0.01. 3 days post-infection, the supernatant of the infected Calu-3 cells was collected and cleared by centrifugation (500 × g, 5 min). To generate high titer viral stock and to eliminate possible influence of cell culture supernatant to the experiment, the viral particles were isolated using an Intact Virus Precipitation Reagent (Thermofisher, 10720D) according to manufacturer’s protocol. The virus pellet was resuspended in fresh cell culture media, aliquoted, and stored at −80 °C prior to use. The titer of the virus stock was determined by TCID50 assay in Vero-E6 cells as previously described and calculated according to the Spearman-Kaerber formula [38]. All experiments involving SARS-CoV-2 virus were performed in a biosafety level 3 (BSL-3) laboratory.

### Isolation of Human Peripheral Blood Mononuclear Cells (PBMCs)

Human blood samples were obtained from consenting volunteers, in accordance with local ethic committee’s approval. PBMCs were isolated from EDTA-anticoagulated whole blood by density centrifugation. Briefly, EDTA blood was diluted 1:1 with PBS. 15 ml of diluted EDTA-blood was transferred into a 50 ml tube containing 10 ml of Ficol-Paque (Merck, GE17-1440–02) and centrifuged at 400 × g for 20 min. The PBMC layer was isolated and washed with PBS. Isolated PBMCs were frozen in 10% DMSO and 90% heat-inactivated FBS and stored at −150 °C before use.

### Infection of HPMC and HAoEC with SARS-CoV-2 in Static Conditions

HPMC and HAoEC were seeded on a chamber slide at a seeding density of 50,000 cells/well 1 day prior to infection. SARS-CoV-2 virus stock was diluted to the desired MOI with complete media and 200 μl of diluted virus stock was used to infect the ECs at MOI of 1. As controls, untreated cells were used. The cells were incubated in a humidified incubator at 37 °C and with 5% CO_2_ for 48 h. Thereafter, the inoculum was removed, and the cells were washed three times with PBS, followed by paraformaldehyde (PFA) fixation for immunofluorescence analysis.

### Culturing and Activation of Endothelial Cells in a Microfluidic System

To culture the ECs in a microfluidic system, 100,000 HPMC and HAoEC in 100 μl of growth media were seeded in a μ-slide VI 0.4 (Ibidi, 80606) overnight. Prior to seeding, the μ-slide was coated with 100 μl of 12.5 μg/ml fibronectin (Merck, FC010) for at least 30 min at 37 °C. The day after, each channel was connected to a peristaltic pump (Gilson, Minipuls 3) using sterile silicon tubings. Cascade media, which consists of Medium 200 (Gibco) supplemented with 10% FBS, 1% glutamine, 1%BSA, and 4% dextran (Sigma Aldrich, 31390), was used as the flow medium. The laminar shear stress was adjusted to 10 dyn/cm^2^ and maintained for 72 h in a humidified incubator at 37 °C and with 5% CO_2_. The media was refreshed every day.

After 72 h of EC culture under flow conditions, HPMC and HAoEC were perfused with 4 ml of cascade media containing 1 μg/ml of recombinant SARS-CoV-2 spike protein derived from the original strain (spike extracellular domain (ECD)-His-Tag (Val16-Pro1213), accession # P0DTC2, Genscript, Z03481), or 1 ng/ml recombinant human TNF-α (RnD Systems, 210-TA-020) for 24 h. Thereafter, the cells were washed with cascade medium by continuous perfusion of 4 ml medium. The medium was then refreshed, and the cells were further cultured under flow conditions for a total of 96 h after activation. A schematic overview of the experiment is given in Supplementary Fig. 3.

### Immunofluorescence Analysis

For immunofluorescence staining, cells grown under static or under microfluidic flow were fixed with 4% PFA for 15 min at room temperature (RT). The cells were washed with PBS, followed by a blocking step at RT with PBS containing 3% BSA. Next, cells were incubated with primary antibody diluted in the antibody solution (1% BSA, 0.05% Tween 20 in PBS) for 2 h at RT, or overnight at 4 °C. Subsequently, cells were stained using a goat polyclonal antibody against human VE-Cadherin (RnD systems, AF938), mouse antibody against ICAM1 (Abcam, ab2213), and E-Selectin (Sigma, S9555) to visualize the endothelial junctions and activation markers. To stain for the viral nucleoprotein and the presence of double-stranded RNA, a SARS-CoV-2 cross-reacting rabbit antibody against SARS-CoV Nucleoprotein (Rockland 200–401-A50) and a mouse antibody against double-stranded RNA (Scicons, J2) were used, respectively. Subsequently, cells were incubated with secondary antibody labeled with fluorophores: Donkey anti-goat IgG (H + L) conjugated with Alexa Fluor 633 (Invitrogen, A21082), donkey anti-mouse IgG (H + L) conjugated with Alexa Fluor 488 (Invitrogen, A32766), and donkey anti-rabbit IgG (H+L) conjugated with Alexa Fluor 568 (Invitrogen, A10042). All secondary antibodies were diluted in the antibody solution and the incubation was performed for 1.5 h at RT. The cells were counterstained with DAPI to visualize nuclei. Cells were imaged using a 20 × objective on a Zeiss LSM 980 confocal microscope. Figures were analyzed using ImageJ (version 2.14.0/1-54f) and assembled using FigureJ package [39, 40]. Brightness and contrast were adjusted identically to the corresponding controls. Quantification of the immunofluorescence signal was done by measuring the area above threshold on six images acquired randomly for each channel.

### Cytokines and Chemokines Analysis

Perfusion media at 24 h and 96 h were collected and stored at −20 °C prior to analysis. The cytokines and chemokines released by ECs in the microfluidic media were simultaneously measured using the Bio-Plex Pro™ Human Chemokine Panel 40-Plex kit (Biorad Laboratories, 171AK99MR2) according to the manufacturer’s protocol. The fluorescence signal was measured using the Bio-Plex 3D Suspension Array System. Chemokines expression fold was calculated as a ratio between spike- and TNF-α-treated versus mock.

### Bulk-RNA Sequencing and Data Analysis

Total cellular RNA from mock-, spike-, and TNF-α-activated HAoEC and HPMC cultures were isolated using the NucleoSpin RNA kit (Macherey Nagel, 740955) according to the manufacturer’s guidelines. The total RNA was quantified with a Quantifluor RNA system (Promega, E3310) according to the manufacturer’s protocol. Bulk RNA barcoding and sequencing (BRB-seq) was performed by Alithea Genomics, as described previously [41]. Briefly, a total of 200 ng of cellular RNA from four independent biological replicates was used for the generation of BRB-seq libraries, followed by sequencing on an Illumina HiSeq 4000 platform to a depth of approximately 5 million raw reads per sample. Demultiplexing, alignment, and count matrix generation was performed using the BRB-seq pipeline.

The libraries were normalized and log1P transformed using Seurat package (version 4.3.0). Differential gene expression analysis was performed with FindMarkers() function in Seurat (test.use = "DESeq2") with an adjusted p-value cutoff of 0.05 and absolute logFC cutoff of 0.25. Visualizations of overlapping DEGs amongst samples were performed using UpSetR (version 1.4.0). Additionally, an online tool provided by VIB/UGent (https://bioinformatics.psb.ugent.be/webtools/Venn/) was used. Pathway enrichment analysis was performed using clusterProfiler (version 4.6.2) with a false discovery cutoff of 0.05. KEGG analysis was performed using pathview (version 1.38.0). Further data analysis and visualization was performed using a variety of additional packages in R.

### Microfluidic PBMC Adhesion Assays

Characterization of PBMC adhesion was performed as previously described [42]. To trace PBMC adhesion on ECs using a microscopy technique, frozen PBMCs were thawed, washed with PBS, and labeled with CFSE (Thermofisher, C34554) according to the manufacturer’s protocol. After the activation of the ECs in the microfluidic channels as described above, the cells were stained for nuclei by perfusing the cells with media spiked with Hoechst 3342 (Tocris, 23,491–52-3). The ECs in the microfluidic channels were subsequently perfused with 1 million/ml CFSE-labelled PBMCs at a shear stress of 0.2 dyn/cm^2^. Using confocal microscopy (Zeiss LSM 980), images were acquired every 3 s for 20 min during PBMC perfusion. PBMC adhesion to the surface of the ECs was defined as the number of cells immobilized to the surface of the ECs for at least 3 frames. Cell counting was done manually for each time-lapse recording.

### Microfluidic Coagulation Assays

Coagulation assays to measure the clotting time after activation of the ECs in the microfluidic system was performed as previously described [42]. Cells were perfused with Hoechst 3342 to visualize the nuclei. Thereafter, the cells were washed with calcium- and magnesium-free PBS and subsequently perfused with human citrated plasma spiked with 15 μg/ml AF488 labeled human fibrinogen (Thermofisher, F13191). The plasma was recalcified with 25 mM CaCl_2_ immediately before imaging. Perfused channels were imaged every 5 s up to 20 min using a time-lapse program on a confocal microscope (Zeiss LSM 980). The coagulation time was determined when complete occlusion of the channel occurred and saturated signal of fibrinogen 488 appeared on the acquisition frame.

### Statistical Analysis

All data are presented as the mean ± standard deviation (SD). The statistical analysis was performed using GraphPad Prism 10 (version 10.0.2). For multiple comparisons ordinary one-way ANOVA followed by Fisher LSD test were performed. All experiments were independently replicated at least three times.

## Results

### SARS-CoV-2 Spike Protein Triggered Prolonged Expression of ICAM1 in Aortic and Lung Microvascular Endothelial Cells

To characterize the extent of vascular inflammatory effects of SARS-CoV-2 spike protein, we utilized HPMC and HAoEC as representatives of two types of ECs from different anatomical regions. In line with earlier studies, we showed that these ECs cannot be productively infected by active SARS-CoV-2 virus (Supplementary Fig. 1) [16]. Neither nucleoprotein (NP) nor double-stranded RNA (dsRNA) can be detected on HPMC and HAoEC 48 h after exposure to SARS-CoV-2 at a multiplicity MOI of 1, in contrast to control cells, Vero-E6. Nevertheless, we observed that both HPMC and HAoEC can be activated with the whole SARS-CoV-2 virus 48 h after exposure, indicated by the increased expression of the cell adhesion molecule ICAM1 (Supplementary Fig. 2). Moreover, as circulating spike protein in COVID-19 patients’ blood has been reported, we also incubated ECs with spike protein (recombinant spike extracellular domain-His-tag protein, original Wuhan strain) and observed increased ICAM1 expression at 24 h (Supplementary Fig. 2) [43–45]. As this result indicated that the whole virus particle is not needed to elicit a vascular response, we decided to use SARS-CoV-2 spike protein to serve as a proxy for studying the effect of SARS-CoV-2 on the vascular endothelium.

To better replicate the physiological conditions of the vascular endothelium, we cultured and treated the ECs under flow conditions (shear stress = 10 dyn/cm^2^). We allowed the cells to grow for 72 h, after which they were exposed to 1 μg/ml of recombinant SARS-CoV-2 spike protein for 24 h, representing the concentration of spike protein concentration used in previous studies, as well as the observed circulating spike protein in COVID-19 patient serum [18, 43–46]. As controls, we included treatment with 1 ng/ml TNF-α. To evaluate the more long-term effect, we stopped the treatment after 24 h and further cultured the ECs to 96 h post-treatment. A schematic overview of the experimental setup for HPMC and HAoEC under flow is depicted in Supplementary Fig. 3.

**Fig. 1:**
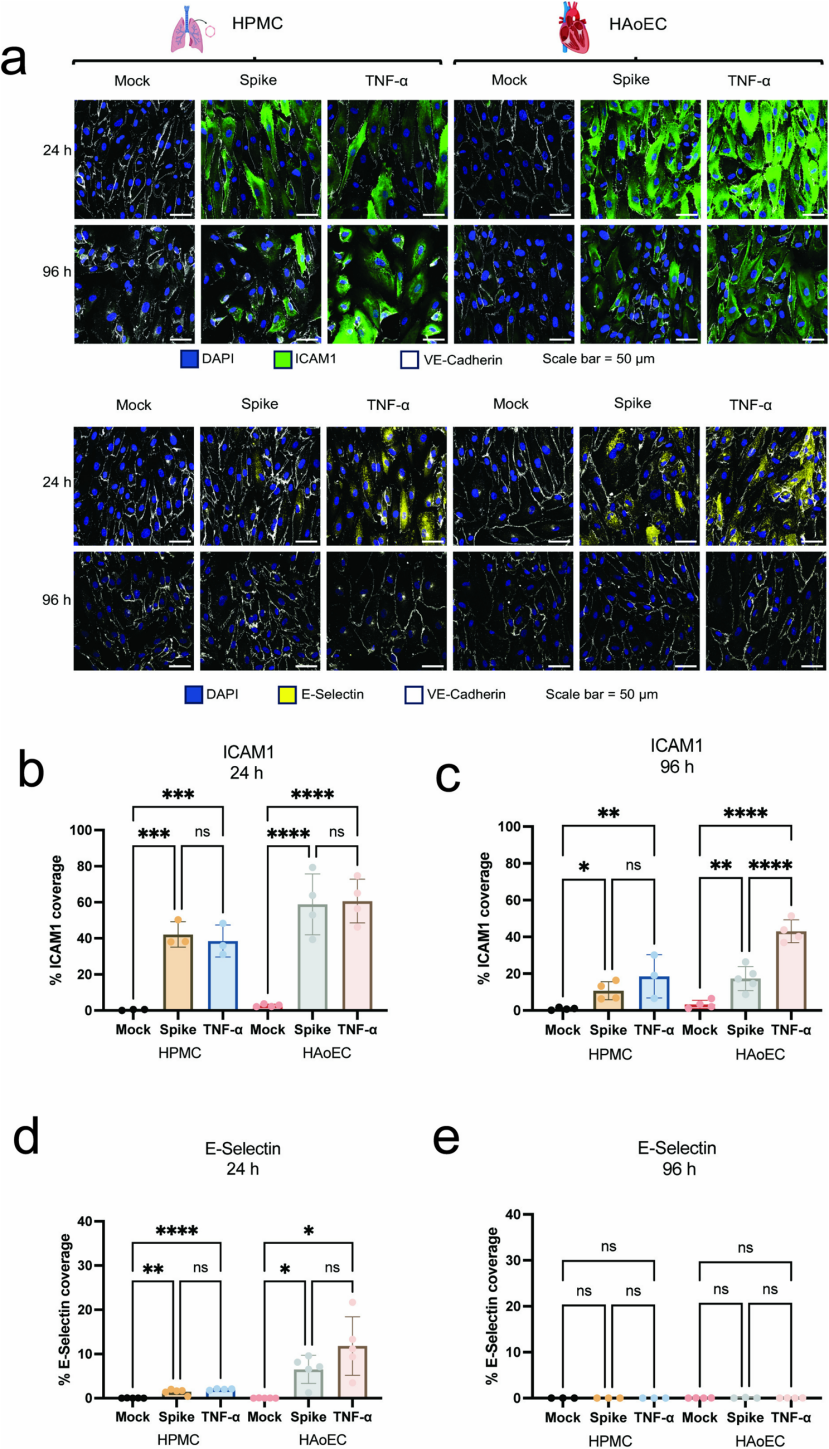
SARS-CoV-2 spike protein-activated endothelial cells exhibit prolonged ICAM1 expression. Human pulmonary microvascular endothelial cells (HPMC) and human aortic endothelial cells (HAoEC) grown under flow conditions (10 dyne/cm^2^) and treated with 1 μg/ml SARS-CoV-2 spike protein, 1 ng/ml TNF-α, or remained untreated for 24 h. The cells were washed after the activation and further cultured for a total of 96 h post-activation. At the indicated time point, cells were fixed and stained for ICAM1 (green), E-Selectin (yellow), VE-Cadherin (white), and nuclei (blue) (**a**). Figures depict representative images. Quantification of the coverage of ICAM1 (**b**, **c**) as well as E-Selectin (**d**, **e**) at 24 h and 96 h post-treatment, respectively, were obtained from at least three biological replicates. Statistical analysis was done using one-way ANOVA with multiple comparisons

We saw that treatment of both HPMC and HAoEC with SARS-CoV-2 spike led to the expression of the cellular adhesion molecules ICAM1 and E-Selectin. We observed a significant induction of ICAM1 (p-value = 0.0001 for HMPC, < 0.0001 for HAoEC) and E-Selectin (p-value = 0.0069 for HPMC, 0.01 for HAoEC) expression at 24 h and post-activation with SARS-CoV-2 spike compared to untreated controls (Figs. 1a, b, and d). At 96 h post-treatment, we saw that a significant ICAM1 expression can still be detected on both HPMC and HAoEC (p-value = 0.03 and 0.0026, respectively, Figs. 1a, c). The expression of E-Selectin, however, was not detectable anymore at 96 h post-treatment (Figs. 1a, e). Treatment with TNF-α had a similar effect as SARS-CoV-2 spike on ICAM1 expression after 24 h (Fig. 1b) and E-Selectin expression after 24 h and 96 h (Figs. 1d, e). In contrast, expression of ICAM1 after 96 h was higher with TNF-α treatment on HAoEC (Fig. 1c). Taken together, these results suggest that the expression of immune cell adhesion molecules by SARS-CoV-2 activation, especially ICAM1, might continue even after the infection has been cleared.

### SARS-CoV-2 Spike Activation Triggers Cytokine and Chemokine Release

**Fig. 2:**
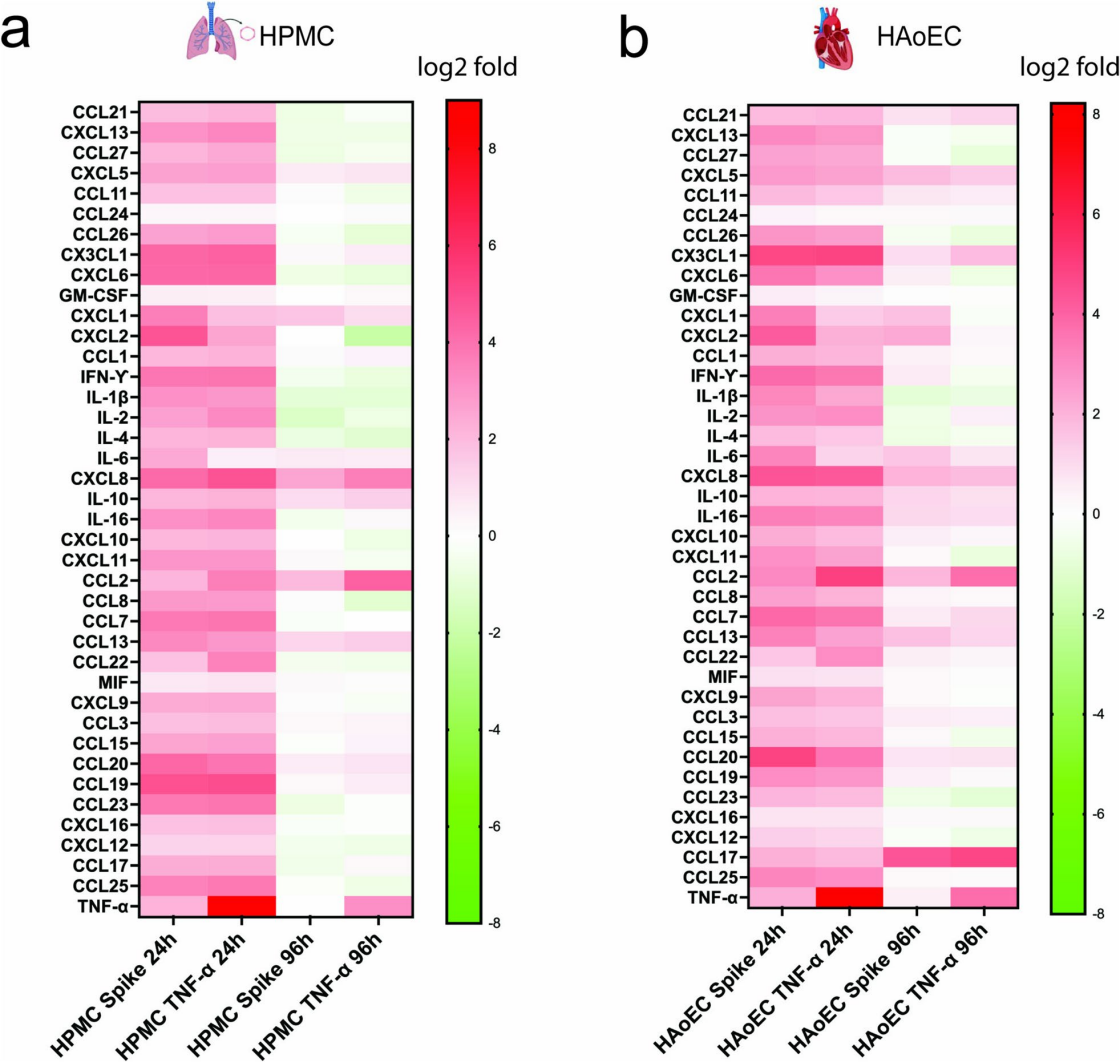
SARS-CoV-2 spike triggers cytokine and chemokine releases from the endothelial cells. Expression of various cytokines and chemokines in human pulmonary microvascular cells (HPMC, **a**) and human aortic endothelial cells (HAoEC,** b**) were measured using a commercial bead-based multiplex ELISA and shown in log2 fold induction over mock at 24 h and 96 h post-treatment with 1 μg/ml SARS-CoV-2 spike protein or 1 ng/ml TNF-α

To see the profile of the cytokine and chemokine releases by human ECs due to SARS-CoV-2 spike protein treatment, we measured the level of cytokines and chemokines in the perfusion media 24 h and 96 h after treatment using a Luminex-type assay. For HPMC (Fig. 2a) and HAoEC (Fig. 2b), we observed cytokine and chemokine changes due to treatment with SARS-CoV-2 spike protein or TNF-α at both time points. At 24 h, most cytokines and chemokines were upregulated to various degrees. For both HPMC and HAoEC, several cytokine and chemokine levels in SARS-CoV-2 spike-treated ECs were increased to a comparable level as with TNF-α (Figs. 2a, b). Some cell type- and treatment-specific changes were also observed. Notably, cytokines and chemokines associated with microbial infection, such as CXCL1 and CXCL2, and proinflammatory cytokine IL-6 were expressed at a considerably higher level with SARS-CoV-2 spike than with TNF-α at 24 h (Figs. 2a, b). An increased TNF-α release in SARS-CoV-2 treated ECs was observed at 24 h. At 96 h post-treatment we saw that the overall induction of cytokine and chemokine expression was reduced. However, some cytokines and chemokines (for instance CCL2, CCL13, and CXCL8, and IL-10 for both cells, CCL17 for HAoEC) were still detectable at a relatively high level.

### SARS-CoV-2 Spike Triggers a Procoagulant State of ECs and the Attachment of PBMCs to the Surface of the ECs

**Fig. 3:**
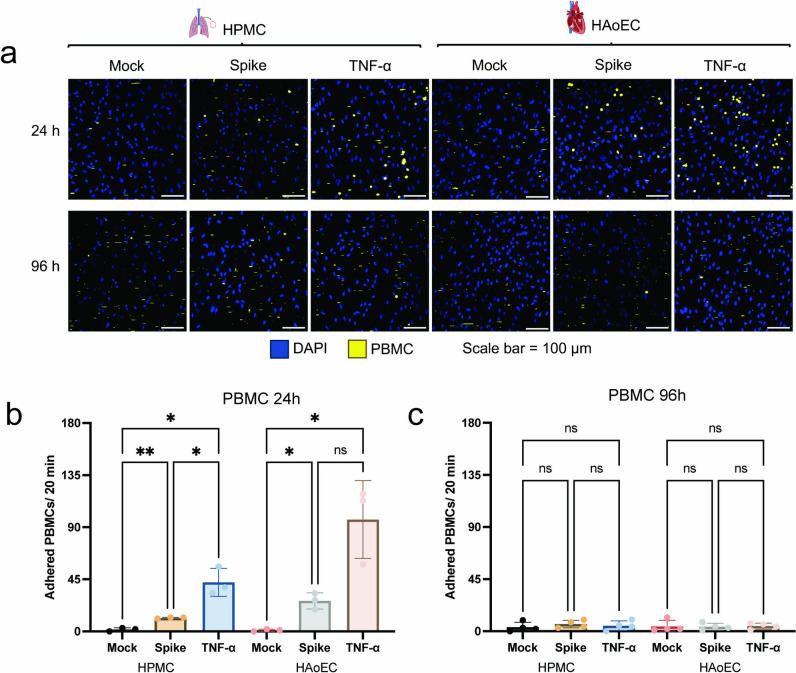
SARS-CoV-2 spike activation leads to increased leucocyte adhesion to the surface of the endothelium. After 24 h activation with 1 μg/ml SARS-CoV-2 spike protein or 1 ng/ml TNF-α, HPMC and HAoEC were perfused with fluorescently labeled PBMCs for 20 min. Representative images from the time-series recording of PBMC adhesion to HPMC and HAoEC 24 h and 96 h post-treatment (**a**). Adhered PBMCs (defined as PBMCs that adhered for more than 3 s in the frame) to HPMC and HAoEC per 20 min at 24 h (**b**) and 96 h (**c**) post-treatment. Statistical analysis was done using one-way ANOVA with multiple comparisons

Next, we sought to evaluate whether the activation of the human endothelium by SARS-CoV-2 spike protein leads to an increased interaction of the ECs with innate immune cells. We performed leucocyte binding assays by perfusing the activated ECs with fluorescently labeled PBMCs and quantified the number of adhering PBMCs using time-series confocal microscopy imaging. Adhered PBMCs were defined as cells attached for two or more frames in a time-series recording that captured the image every 3 s. Using this assay, we saw that treatment with SARS-CoV-2 spike protein induced significant binding of leucocytes to HPMC and HAoEC at 24 h (p-value = 0.0035 and 0.023, respectively), and even more so with TNF-α treatment (Figs. 3a, b). At 96 h post-treatment, however, we no longer observed PBMC binding on both types of ECs for either treatment (Figs. 3a, c).

**Fig. 4:**
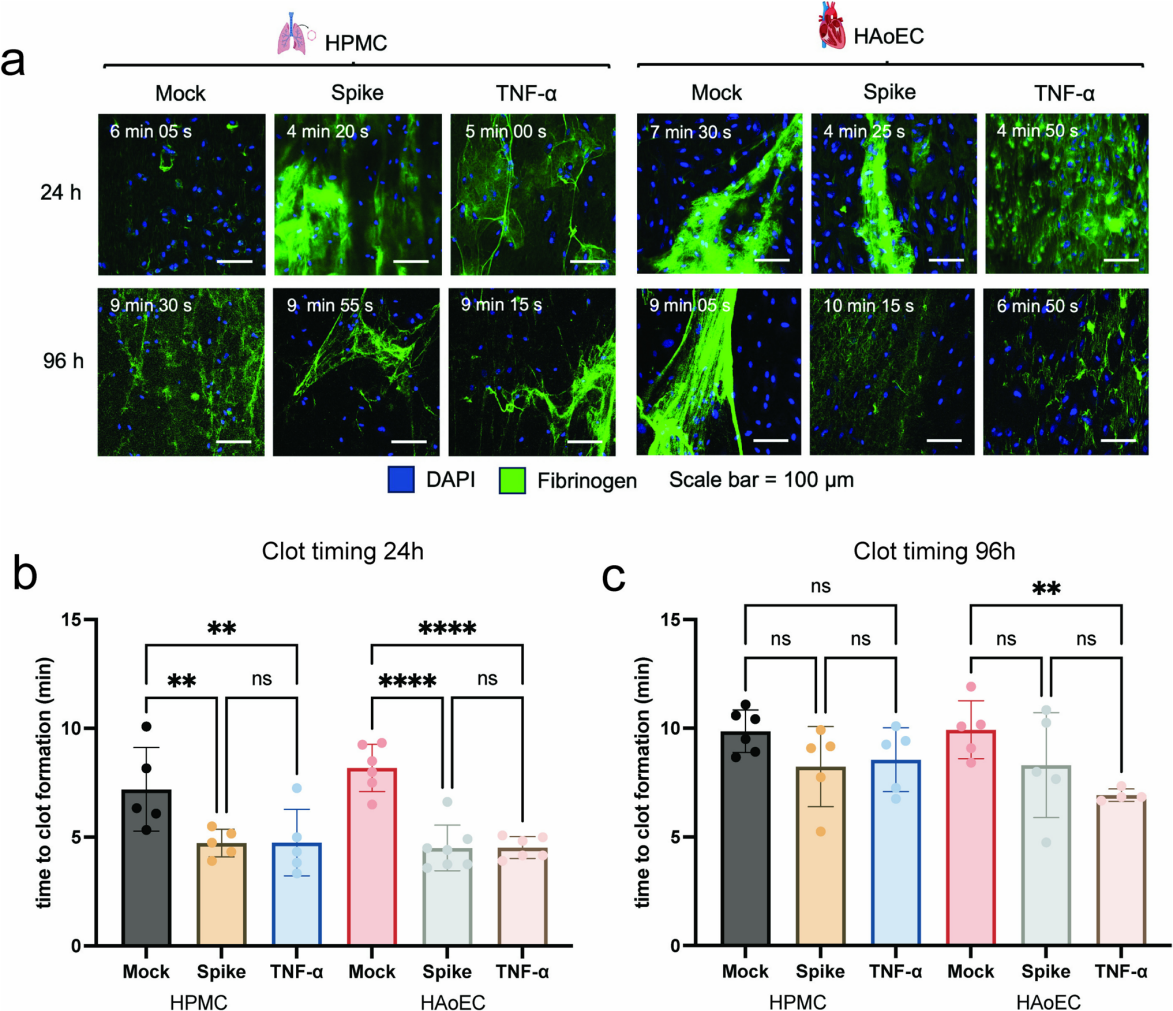
SARS-CoV-2 spike induces a procoagulant state of the human endothelium. SARS-CoV-2 spike and TNF-α-treated HPMC and HAoEC were perfused with recalcified citrated human plasma spiked with fluorescently labeled fibrinogen. Representative images of clot formation HPMC and HAoEC 24 h and 96 h post-treatment (**a**). Time to clot formation was determined from the time series imaging, defined as the time when complete occlusion of the channel and the formation of saturated fluorescence signal from the fluorescently labeled fibrinogen were observed. Clotting time for both ECs at 24 h (**b**) and 96 h (**c**) after treatment. One-way ANOVA with multiple comparisons was used for statistical analysis

We then analyzed the influence of SARS-CoV-2 spike activation on clot formation on the ECs. After activating the cells with SARS-CoV-2 spike or TNF-α, we perfused the ECs with recalcified human citrate plasma spiked with fluorescently labeled fibrinogen and used time-series microscopy imaging to record the clot formation time. As shown in Figs. 4a and b, we observed a significant decrease in clot formation time, indicative of a procoagulant state in HPMC and HAoEC, 24 h post-treatment with SARS-CoV-2 spike protein, and also with TNF-α. At 96 h post-treatment, the reduction of clot formation time was less pronounced and only statistically significant for HAoEC treated with TNF-α (Figs. 4a, c).

### Transcriptional Dynamics of SARS-CoV-2 Spike Activation of the Human Endothelium

Given the observed influence of SARS-CoV-2 spike protein on EC activation, we sought to gain further insight into the influence of the spike protein on EC dysfunction by transcriptional analysis. We extracted the total cellular mRNA from mock-, SARS-CoV-2 spike-, and TNF-α-treated HPMC and HAoEC 24 h and 96 h post-treatment. RNA sequencing was performed using the bulk RNA barcoding and sequencing (BRB-seq) pipeline [41]. 653 unique, differentially expressed genes (DEGs) were identified across samples by performing pairwise comparisons between each treatment and the mock counterpart for each cell type and time point (adjusted p-value < 0.05, absolute logFC > 0.25). For spike- and TNF-α-treated HAoEC 24 h post-activation, 215 and 352 DEGs were identified, respectively. For HPMC, 320 and 342 DEGs were identified for treatment with spike and TNF-α after 24 h, respectively. DEGs were also identified at 96 h, albeit significantly lower (spike-treated HAoEC = 20, TNF-α-treated HAoEC = 4, spike-treated HPMC = 33, TNF-α-treated HPMC = 17), confirming the prolonged effect on EC gene expression profiles. All the DEGs identified in all comparisons are listed in Supplementary Table 1.

**Fig. 5:**
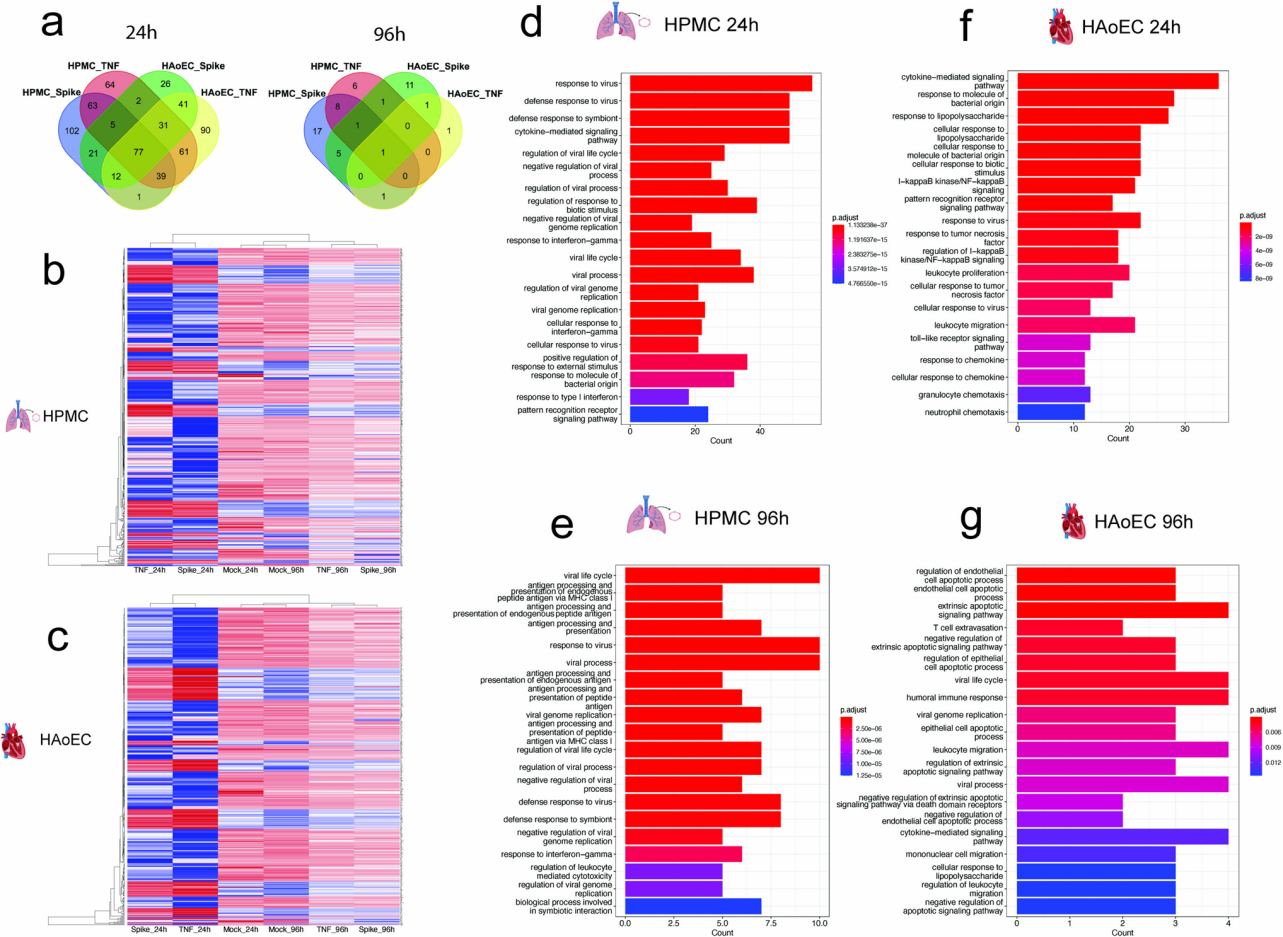
Transcriptional change due to SARS-CoV-2 activation in the human endothelium. Venn diagram depicting overlapping differentially expressed genes (DEGs) for HPMC and HAoEC 24 h and 96 h post-treatment with SARS-CoV-2 spike and TNF-α (**a**). Heatmap of hierarchical clustering of all DEGs identified for HPMC (**b**) and HAoEC (**c**). Expression level was shown as the log-transformed of the average normalized counts from four independent replicates. Biological process enrichment analysis results for upregulated DEGs in HPMC (**d, e**) and HAoEC (**f, g**) 24 h and 96 h post-treatment with SARS-CoV-2 spike

More DEGs were shared among all treatments at 24 h post-treatment than at 96 h (Fig. 5a, Supplementary Figs. 4a and b). For instance, 59 upregulated and 8 downregulated DEGs were shared among all conditions exclusively at 24 h (Supplementary Figs. 4a, b). At 96 h, only 1 DEG was shared among conditions. Unique DEGs for HPMC and HAoEC were also identified, highlighting distinct transcriptional responses of the two ECs stemming from different anatomical locations. Hierarchical clustering of all DEGs identified for HPMC and HAoEC also showed a distinct expression of several gene clusters in SARS-CoV-2 treated ECs in comparison to those with TNF-α treatment (Figs. 5b, c).

To examine the pathways involved in the vascular inflammatory effect of the SARS-CoV-2 spike protein, we performed a pathway-enrichment analysis on DEGs of HPMC and HAoEC at 24 h and 96 h time points, respectively (Fig. 5d-g, Supplementary Figs. 4c and 5). We saw that 24 h after treatment with SARS-CoV-2 spike protein, upregulation of cytokine-mediated signaling is observed for HMPC and HAoEC, similarly to the treatment with TNF-α (Figs. 5d and f, Supplementary Fig. 5). Pathways associated with responses to viruses are more enriched for HPMC, while pathways associated with responses to other pathogens were seen in HAoEC. Pattern recognition receptor signaling pathway, toll-like receptor signaling, and NF-kB signaling were also enriched. Furthermore, pathways associated with cellular development were downregulated for HPMC at 24 h (Supplementary Fig. 4c). At 96 h, upregulation of pathways associated with virus response were still observed in the spike-treated HPMC (Fig. 5e). In addition, antigen presentation and processing pathways were highly represented, indicating the activation of immunomodulating functions in the ECs. For HAoEC, pathways associated with endothelial apoptosis and responses to virus were among the most significantly upregulated while TGF-β regulation pathways were downregulated (Fig. 5g, Supplementary Fig. 4c). For TNF-α-treated HPMC and HAoEC, pathways associated with antigen processing were upregulated, while pathways associated with endothelial development were downregulated (Supplementary Fig. 5).

### SARS-CoV-2 Spike-Treated Cells Exhibit Sustained Inflammation and Alteration of Antigen Presentation and Coagulation State of the Endothelium at a Later Stage

**Fig. 6:**
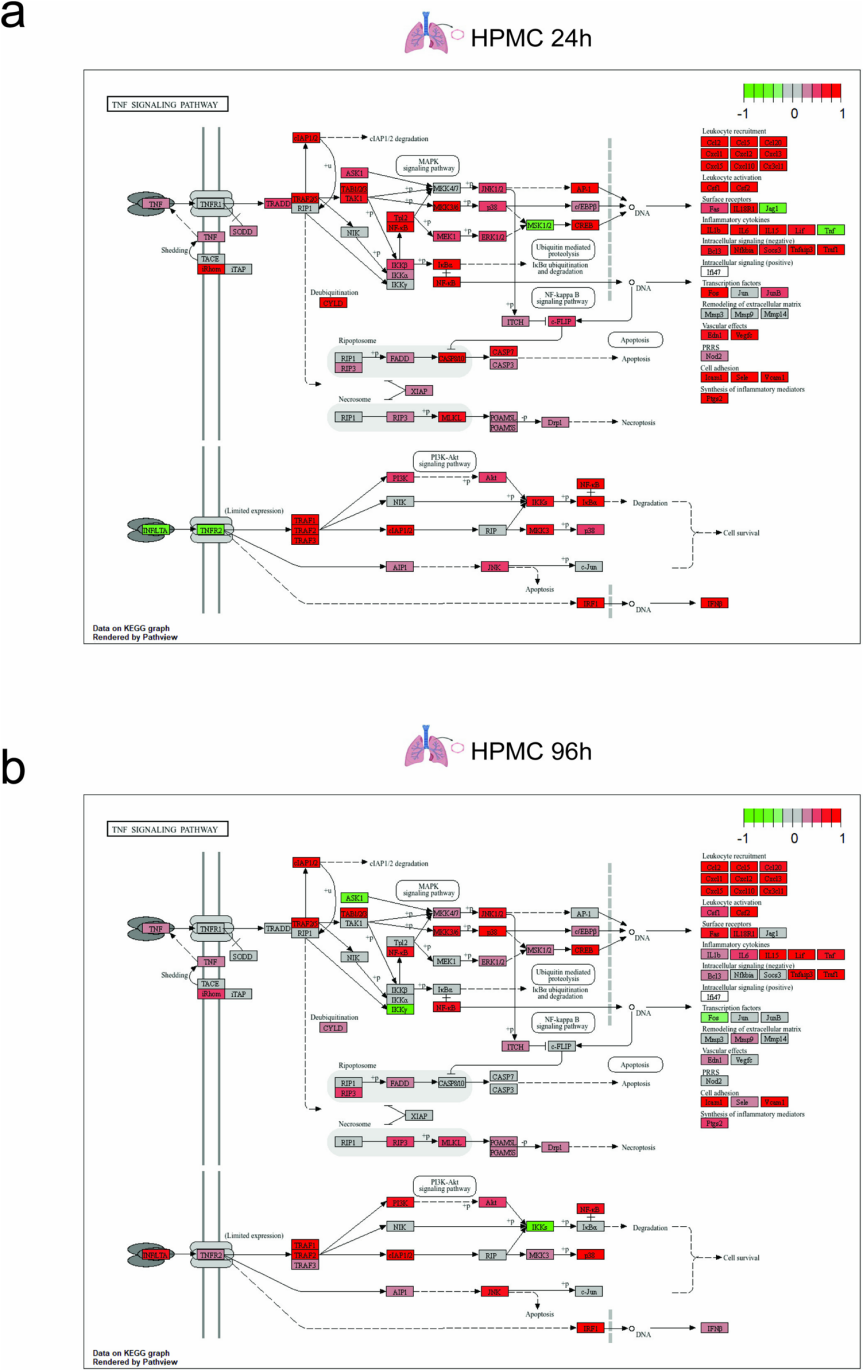
Prolonged expression of genes associated with proinflammatory pathways mediating the vascular inflammatory effect of SARS-CoV-2 spike protein. A segment of the KEGG pathway showing DE genes associated with SARS-CoV-2 spike-activated HPMC at 24 h (**a**) and 96 h (**b**) were significantly enriched in the TNF signaling pathway

**Fig. 7:**
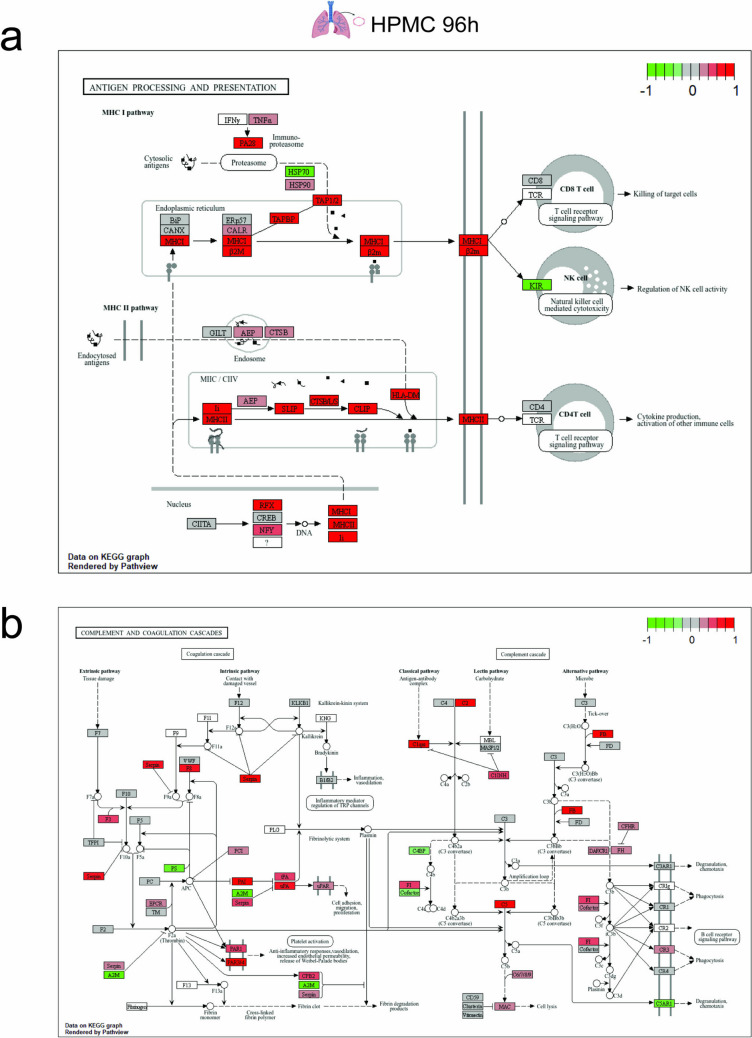
Enrichment of antigen presentation and coagulation cascade at 96 h. KEGG pathway map depicting the enrichment of antigen processing and presentation (**a**) as well as the complement and coagulation pathway (**b**) in the spike-treated HPMC at 96 h

To have a closer look at the prolonged transcriptomic changes associated with the observed pathological conditions in SARS-CoV-2 spike treatments, we analyzed the DEGs of SARS-CoV-2 treated ECs at 96 h with Kyoto Encyclopedia of Genes and Genomes (KEGG) Mapper software. Prolonged expression of individual markers associated with TNF-α signaling for HPMC (Fig. 6a, b) and HAoEC (Supplementary Fig. 6a, b) confirmed a sustained vascular inflammation. MHC class I (HLA-A, HLA-B, and HLA-C) and the transmembrane glycoprotein TAPBP, predominantly mediating the antigen processing and presentation pathway, were also enriched at 96 h (Fig. 7a, Supplementary Fig. 7a). Moreover, the upregulation of Cathepsin S (CTSS) indicates the influence on the MHC class II pathway. The influence on the coagulation pathway was marked by the upregulation of serpin, factor VIII, PAI-1, tPA and uPA, as well as the downregulation of anticoagulant factors such as protein S and alpha-2-macroglobulin (A2M) (Fig. 7b, Supplementary Fig. 7b). Complement-associated factors such as complement factor B, C5, C1 receptor, and MAC were upregulated, whereas a downregulation of C4BP and C5R1 was observed. This suggests that the vascular procoagulant effect of SARS-CoV-2 on the endothelium, although to a lower degree, might last beyond the presence of virus particles.

## Discussion

In this study, we investigated the scope of the vascular inflammatory effect of SARS-CoV-2 spike protein on phenotypic, functional, and transcriptional levels in both lung microvascular and aortic ECs. To provide a more comprehensive insight into the profile of vascular inflammation, we included a comparison with the inflammatory cytokine TNF-α, which is known to induce prolonged activation of ECs [47, 48]. Consistent with previous findings, our study revealed that SARS-CoV-2 spike protein triggered prolonged cell adhesion marker expression and cytokine/chemokine releases, along with increased immune cell binding and formation of a procoagulant state of the ECs [20, 22, 46, 49–51]. We observed a similar degree of vascular inflammation by SARS-CoV-2 spike protein to that with TNF-α. However, distinct gene activation profiles were found for the viral spike protein. We also showed that on the transcriptome level, SARS-CoV-2 spike resulted in sustained inflammation, changes in antigen presentation, and coagulation state of the endothelium. The observed prolonged effects beyond the presence of the spike protein suggests possible long-term consequences of SARS-CoV-2 on the endothelium.

The involvement of ACE2 and vascular infection in mediating vascular inflammation have been described, although findings on this topic are controversial [13, 22, 46, 52–54]. In the present study, we showed that the observed vascular inflammatory effect of SARS-CoV-2 is not caused by active viral replication and most likely not mediated by ACE2, due to lack of ACE2 expression in both types of ECs used (Supplementary Fig. 8) [16, 18, 53, 55]. Although infection of SARS-CoV-2 in the respiratory tract is ACE2-dependent, other spike-binding receptors might be involved in mediating the interaction of SARS-CoV-2 with vascular ECs. Several reports have described that SARS-CoV-2 spike engages glycosaminoglycans (GAGs) on the EC glycocalyx and further binds integrins [18, 29, 56, 57]. Others have described neuropilin-1 as a SARS-CoV-2 spike receptor that mediates the SARS-CoV-2 cellular entry [58]. Additionally, TLR recognition might also be important in mediating the vascular inflammatory effect of SARS-CoV-2 on the vasculature [59]. Therefore, further elucidation and confirmation of the involvement of ACE2 and other receptors in the vascular endothelium inflammation via SARS-CoV-2 spike protein are warranted.

In line with previous findings, we showed that spike-treated ECs express high levels of cellular adhesion markers [29, 46, 52]. Elevated ICAM1 in plasma, which could be released by the damaged endothelium, is also positively correlated with disease severity, as has been observed in COVID-19 patients [60, 61]. Moreover, elevated ICAM1 and other EC adhesion molecules associated with disease severity have been described in chronic cardiovascular diseases, including atherosclerosis and coronary heart disease [62, 63]. Our results also show that the expression of ICAM1 seems to persist beyond the presence of SARS-CoV-2 spike, suggesting a state of sustained inflammation of the ECs. Similarly, several studies have shown increased levels of ICAM1 in serum of patients recovered from COVID-19 [35, 64, 65]. The circulating ICAM1, which could originate from damaged endothelium, may contribute to prolonged inflammation even in recovered and no longer infectious COVID-19 patients, indicating the critical involvement of the endothelium in PASC.

Our results showed similar profiles of cytokine and chemokine expression due to SARS-CoV-2 spike activation on human ECs to those observed in COVID-19 patients [30, 31]. Elevated IL-1β, IL-6, IL-8, IL-10, and IL-17 in plasma is associated with disease severity in COVID-19 patients [66–70]. IL-6 is critical in neutrophil recruitment, increasing vascular permeability, T-cell response regulation, and necrosis, all of which lead to worse clinical outcomes [71–74]. Interestingly, an increased level of the antiinflammatory IL-10 has been reported in severe COVID-19 patients [75–78]. Increased and prolonged IL-10 in the hyperinflammatory conditions of COVID-19 may be related to excessive stimulation and exhaustion of cytotoxic effector CD8 + T cells [79]. Expression of IL-1β, CXCL1, IL-8, and CCL20 could contribute to neutrophil recruitment to the surface of the endothelium, leading to NET-formation and immunothrombosis [50, 69, 70, 80–82]. Chemokines such as CCL8, CXCL2, and CXCL10 can lead to recruitment of monocytes and macrophages to the activated ECs [83–85]. The increased expression of CCL2 also contributes to the amplification of monocyte and macrophage activation [86]. This further highlights the crucial role of ECs in the production of various inflammatory cytokines and chemokines contributing to the cytokine storm and excessive inflammatory response, exacerbating the disease in severe COVID-19 patients [67, 87].

Recruitment of immune cells to the surface of spike-activated ECs can lead to infiltration of inflammatory cells and further damage to the surrounding tissue, which can happen independently of an active infection and in different anatomical regions. Different chemokine expression levels and dynamics over time between HPMC and HAoEC suggest a possible EC origin-specific response. Our RNAseq analysis also highlights the distinct transcriptomic signatures and the pathways associated with SARS-CoV-2 spike activation of HPMC and HAoEC. It is therefore necessary to characterize organ-specific vascular responses from organs that are also affected by COVID-19, such as brain and kidneys.

Amongst the complex aspects of the disease, coagulation dysregulation has been shown as a significant contributor to the morbidity and mortality of COVID-19 infection as well as the pathology of PASC [4, 32, 57, 65, 88]. We have demonstrated that the spike protein can directly increase the procoagulation state of ECs, consistent with the existing data [46, 49, 50]. In addition, the transcriptomic analysis also showed a prolonged disruption of the regulation of the complement and coagulation cascades, reflecting a possible sustained prothrombotic state and increased cardiovascular complications after COVID-19 infection [32, 65]. Increased proinflammatory state and elevated expression of procoagulant factors such as Von Willebrand factor (vWF), reactive oxygen species (ROS), PA-I, and tissue factor (TF) have been shown to contribute to the coagulation dysfunction mediated by SARS-CoV-2 [6, 49, 50, 53]. Our RNAseq data further underscores that SARS-CoV-2 spike alone can trigger an array of pathogen-associated responses, induction of robust proinflammatory states, alteration of EC development, and apoptosis, likely associated with the observed thrombo-inflammatory symptoms in COVID-19 patients [88, 89]. Moreover, prolonged expression of genes associated with proinflammatory pathways and apoptosis could induce persistent endothelial dysfunction and damage. The observed prolonged increase of adhesion molecules and antigen presentation could lengthen the recruitment of immune cells and mediate EC interaction with CD8 + and CD4 + T lymphocytes [90, 91]. 

It is worth noting that we did not see significant changes in the leucocyte binding and clotting time at a later time point in vitro, which could be due to the limitation in the assay sensitivity in our model. It is, therefore, essential to validate the long-term changes due to SARS-CoV-2 spike activation in ECs in a more extensive study, for instance, in animal models or clinical studies involving convalescent COVID-19 patients. Future studies should consider evaluating the consequences of EC activation by SARS-CoV-2 at earlier than 24 h and beyond the indicated time point 96 h to provide a better resolution of the SARS-CoV-2 vascular inflammatory effects. In addition, as this study was conducted using the original strain of SARS-CoV-2, vascular inflammatory effects of other SARS-COV-2 spike variants, especially those with more lethal outcomes or more changes to the spike, should be further characterized [92].

Given the extent of the SARS-CoV-2 spike effect on ECs and its possible contribution to systemic and long-term damage, endothelium-targeted therapies might serve as a promising approach to mitigate the disease outcomes in COVID-19 patients. For instance, preventing the action of pro-inflammatory cytokines and the downstream signaling pathways have been shown to reduce severity and mortality in COVID-19 patients [93–95]. EC-protecting drugs that prevent the activation of ECs, such as glucocorticoids and sulodexide have been shown to elicit positive effects [96, 97]. Drug classes targeted to treat endothelial dysfunction, including renin-angiotensin system (RAS) inhibitors and statins, might be beneficial in preventing and managing systemic complications during SARS-CoV-2 infection [98]. Other than for acute management, EC-targeted therapies hold the potential for addressing long-term sequelae, such as those observed in long COVID. Moreover, as coagulation dysregulation is also one of the essential components of COVID-19 pathophysiology, anticoagulants such as heparin, antiplatelet drugs, fibrinolytic therapies, and direct thrombin inhibitors may help mitigate thrombotic complications [99–102]. Continued research is needed to determine the most appropriate therapeutic strategies, dosing, and duration, taking into account the wide range of inflammatory responses and severity of disease in COVID-19 patients.

In summary, our results provided a detailed and comprehensive characterization of the vascular inflammatory effects of SARS-CoV-2. We showed that the endothelium plays an essential role in determining the outcome of COVID-19 infection, such as vascular inflammation and systemic organ damage during and possibly beyond the acute infection phase. Therapeutic strategies should also consider the extent of SARS-CoV-2 inflammatory effects on the vascular endothelium. Treatments directed to EC protection and prevention of endothelial damage might be essential in the prevention and management of the post-sequelae effect of COVID-19.

## Supplementary Information

Below is the link to the electronic supplementary material.

Supplementary file1 No productive infection of SARS-CoV-2 in human endothelium. Human pulmonary microvascular endothelial cells (HPMC) and human aortic endothelial cells (HAoEC) were grown under static conditions on a chamber slide and infected with purified SARS-CoV-2 virus stock at a MOI of 1 for 48h. As a positive control, Vero-E6 was included. Fixed cells were stained for SARS-CoV-2 Nucleoprotein (red), double-stranded RNA (green), and VE-cadherin (yellow) for endothelial cell markers. Scale bar = 50 µm (TIF 19436 KB).

Supplementary file2 SARS-CoV-2 and SARS-CoV-2 spike protein can activate human endothelium. SARS-CoV-2 infected (upper panel) and SARS-CoV-2 spike-treated (lower panel) HPMC and HAoEC were stained with ICAM1 (green) to visualize the expression of adhesion molecules, indicating the activated state of the endothelium. Scale bar = 50 µm (TIF 35384 KB).

Supplementary file3 Schematic overview of the experimental setup (TIF 10285 KB).

Supplementary file4 Upregulated and downregulated differentially expressed genes (DEGs) of activated endothelial cells. Upset plot depicting the shared upregulated (a) and downregulated (b) DEGs across samples. Bar plots of the most enriched biological processes by downregulated DEGs in the SARS-CoV-2 spike-treated ECs (c) (TIF 46421 KB).

Supplementary file5 Transcriptional changes in TNF-α treated endothelial cells. Barplots of top enriched biological process by upregulated (a) and downregulated (b) DEGs in the TNF-α treated ECs (TIF 39627 KB).

Supplementary file6 A segment of the KEGG pathway showing the enrichment of the TNF signaling pathways by DE genes associated with SARS-CoV-2 spike-activated HAoEC at 24h (a) and 96h (b) (TIF 37335 KB).

Supplementary file7 Enrichment of antigen processing and presentation (a) as well as the complement and coagulation pathway (b) in the SARS-CoV-2 spike-treated HAoEC at 96h (TIF 33668 KB).

Supplementary file8 Expression of ACE2 in HPMC and HAoEC. Expression level was depicted as the log-transformed of the normalized counts of each replicate (TIFF 317 KB).

Supplementary file9 List of DEGs identified in HPMC and HAoEC (XLSX 101 KB).

## Data Availability

Data generated during this study are included in the article. RNAseq data and several basic visualization tools are available online on the Aarhus University repository (https://dreamapp.biomed.au.dk/SARS_EC/).
