# Supplementary figures and images for: Sustained Vascular Inflammatory Effects of SARS-CoV-2 Spike Protein on Human Endothelial Cells
Source: Inflammation. 2024 Dec 31;48(4):2531–47. doi: 10.1007/s10753-024-02208-x (PMC12336097; doi:10.1007/s10753-024-02208-x)

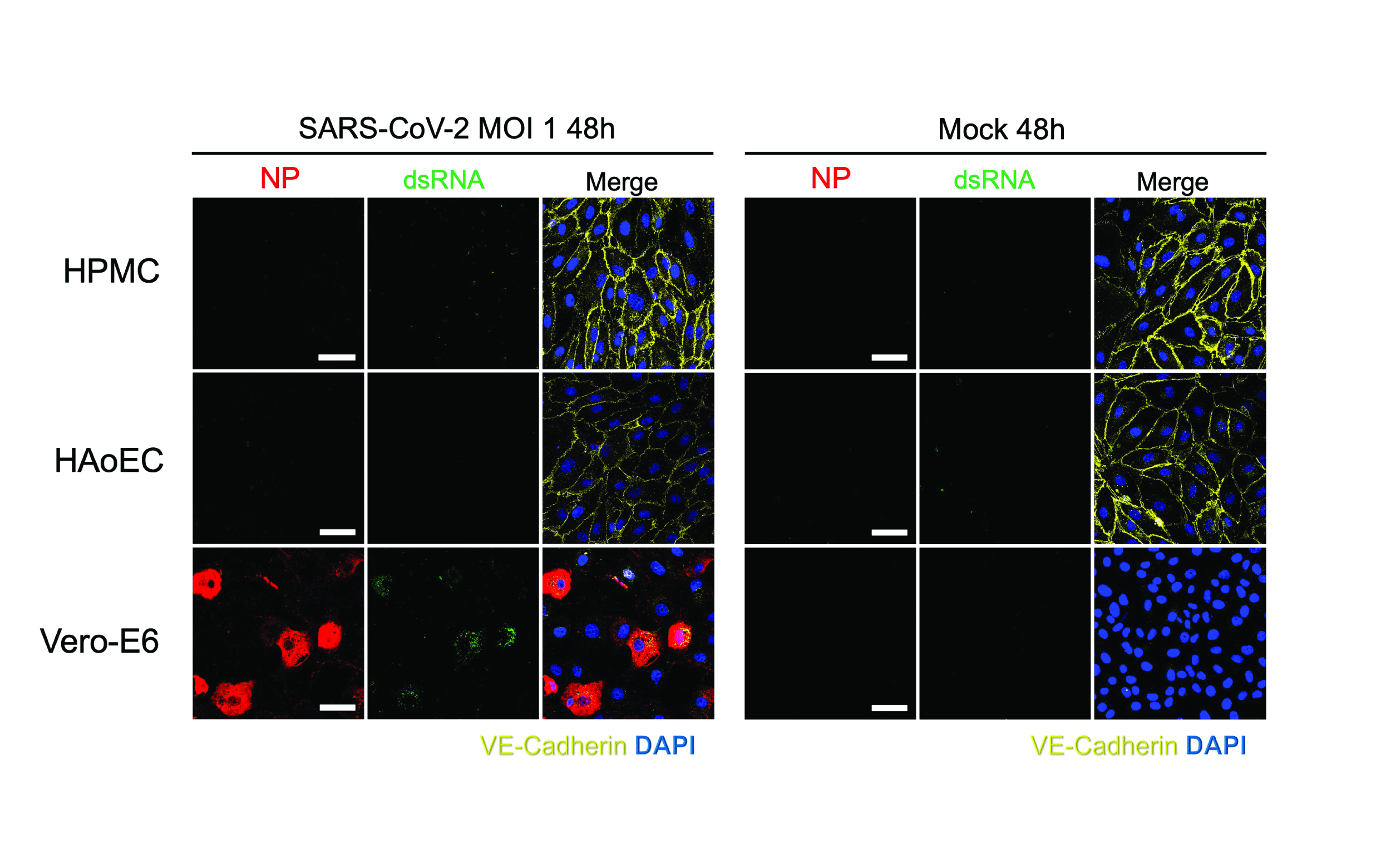

Supplement: Supplementary file 1 — Supplementary file1 No productive infection of SARS-CoV-2 in human endothelium. Human pulmonary microvascular endothelial cells (HPMC) and human aortic endothelial cells (HAoEC) were grown under static conditions on a chamber slide and infected with purified SARS-CoV-2 virus stock at a MOI of 1 for 48h. As a positive control, Vero-E6 was included. Fixed cells were stained for SARS-CoV-2 Nucleoprotein (red), double-stranded RNA (green), and VE-cadherin (yellow) for endothelial cell markers. Scale bar = 50 µm (TIF 19436 KB). [file 10753_2024_2208_MOESM1_ESM.tif]

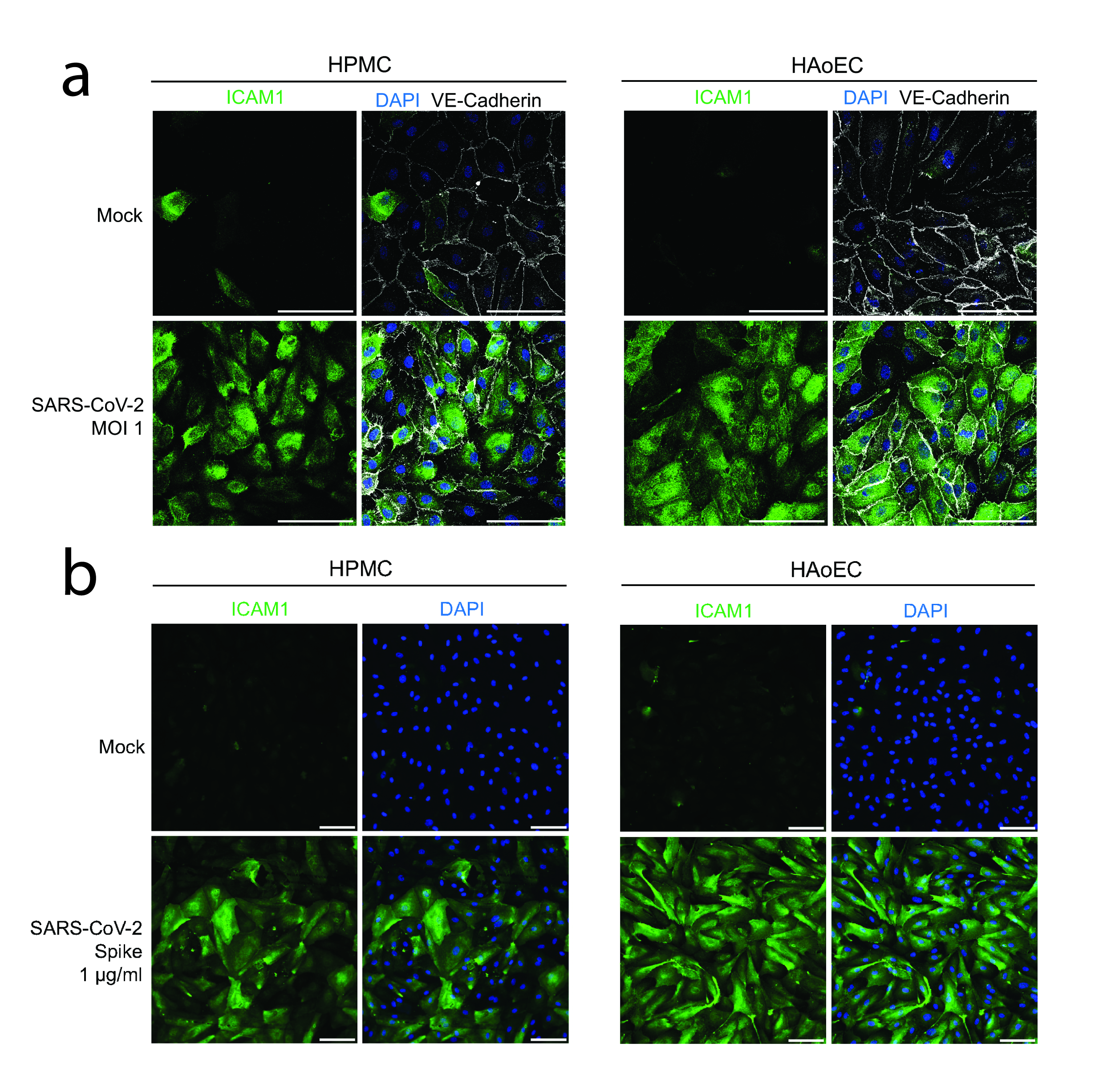

Supplement: Supplementary file 2 — Supplementary file2 SARS-CoV-2 and SARS-CoV-2 spike protein can activate human endothelium. SARS-CoV-2 infected (upper panel) and SARS-CoV-2 spike-treated (lower panel) HPMC and HAoEC were stained with ICAM1 (green) to visualize the expression of adhesion molecules, indicating the activated state of the endothelium. Scale bar = 50 µm (TIF 35384 KB). [file 10753_2024_2208_MOESM2_ESM.tif]

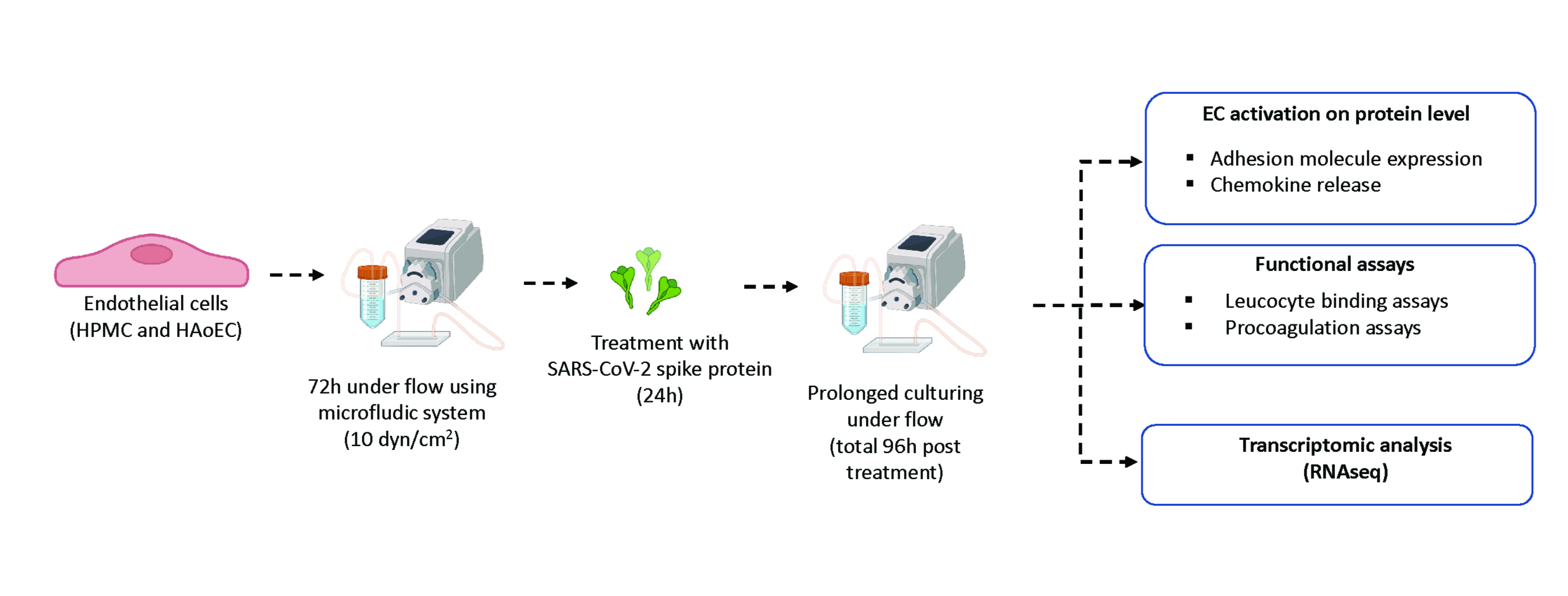

Supplement: Supplementary file 3 — Supplementary file3 Schematic overview of the experimental setup (TIF 10285 KB). [file 10753_2024_2208_MOESM3_ESM.tif]

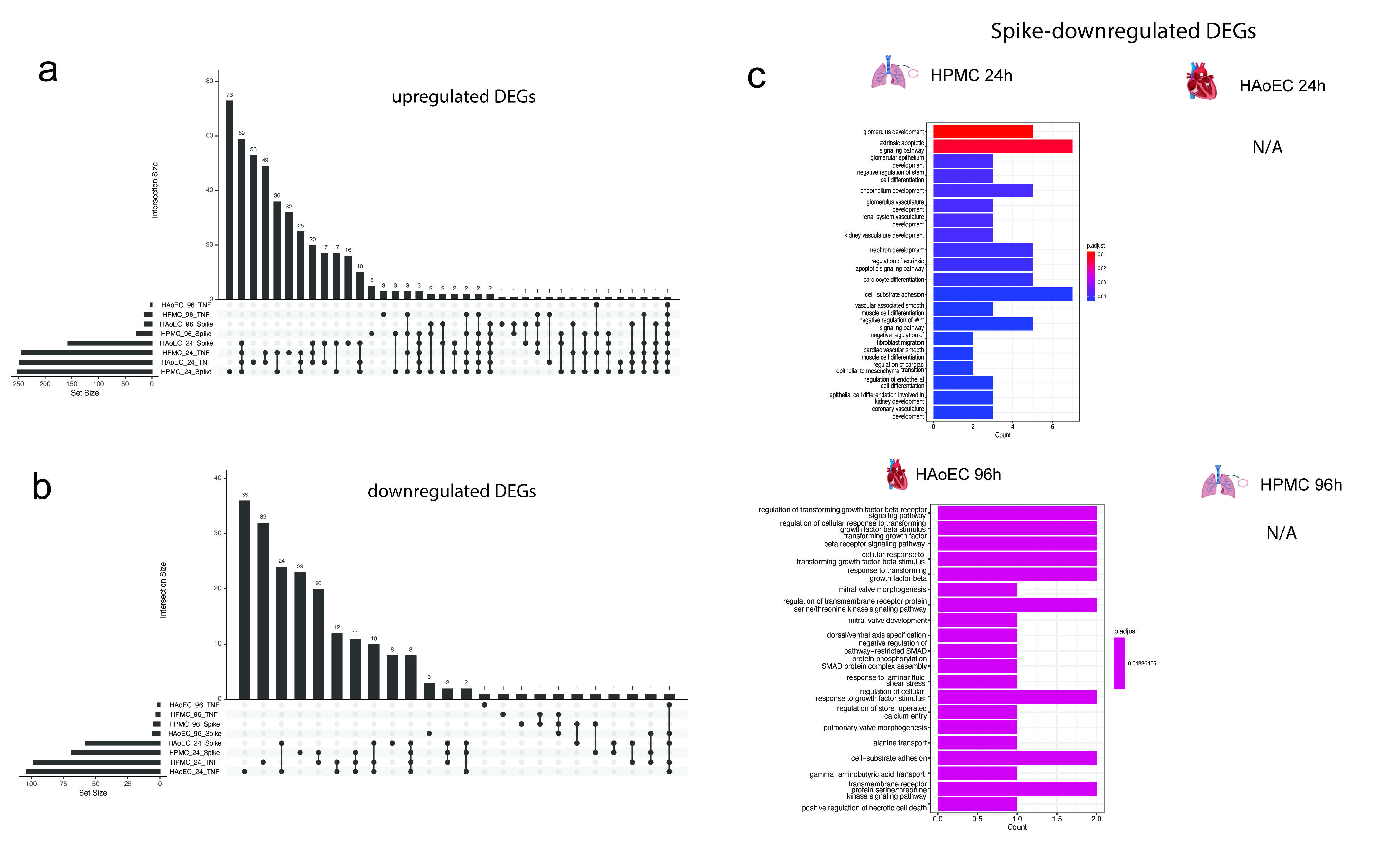

Supplement: Supplementary file 4 — Supplementary file4 Upregulated and downregulated differentially expressed genes (DEGs) of activated endothelial cells. Upset plot depicting the shared upregulated (a) and downregulated (b) DEGs across samples. Bar plots of the most enriched biological processes by downregulated DEGs in the SARS-CoV-2 spike-treated ECs (c) (TIF 46421 KB). [file 10753_2024_2208_MOESM4_ESM.tif]

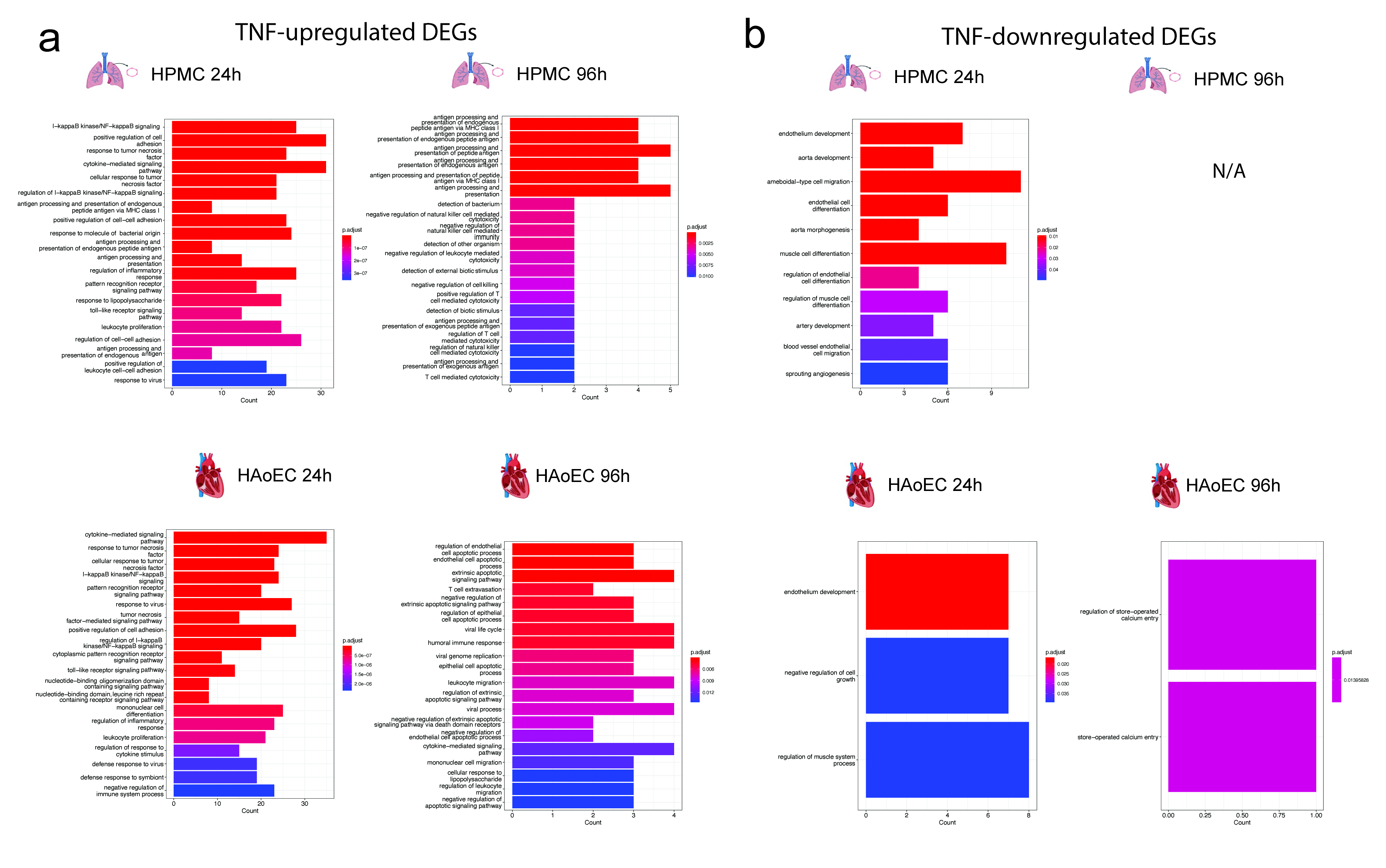

Supplement: Supplementary file 5 — Supplementary file5 Transcriptional changes in TNF-α treated endothelial cells. Barplots of top enriched biological process by upregulated (a) and downregulated (b) DEGs in the TNF-α treated ECs (TIF 39627 KB). [file 10753_2024_2208_MOESM5_ESM.tif]

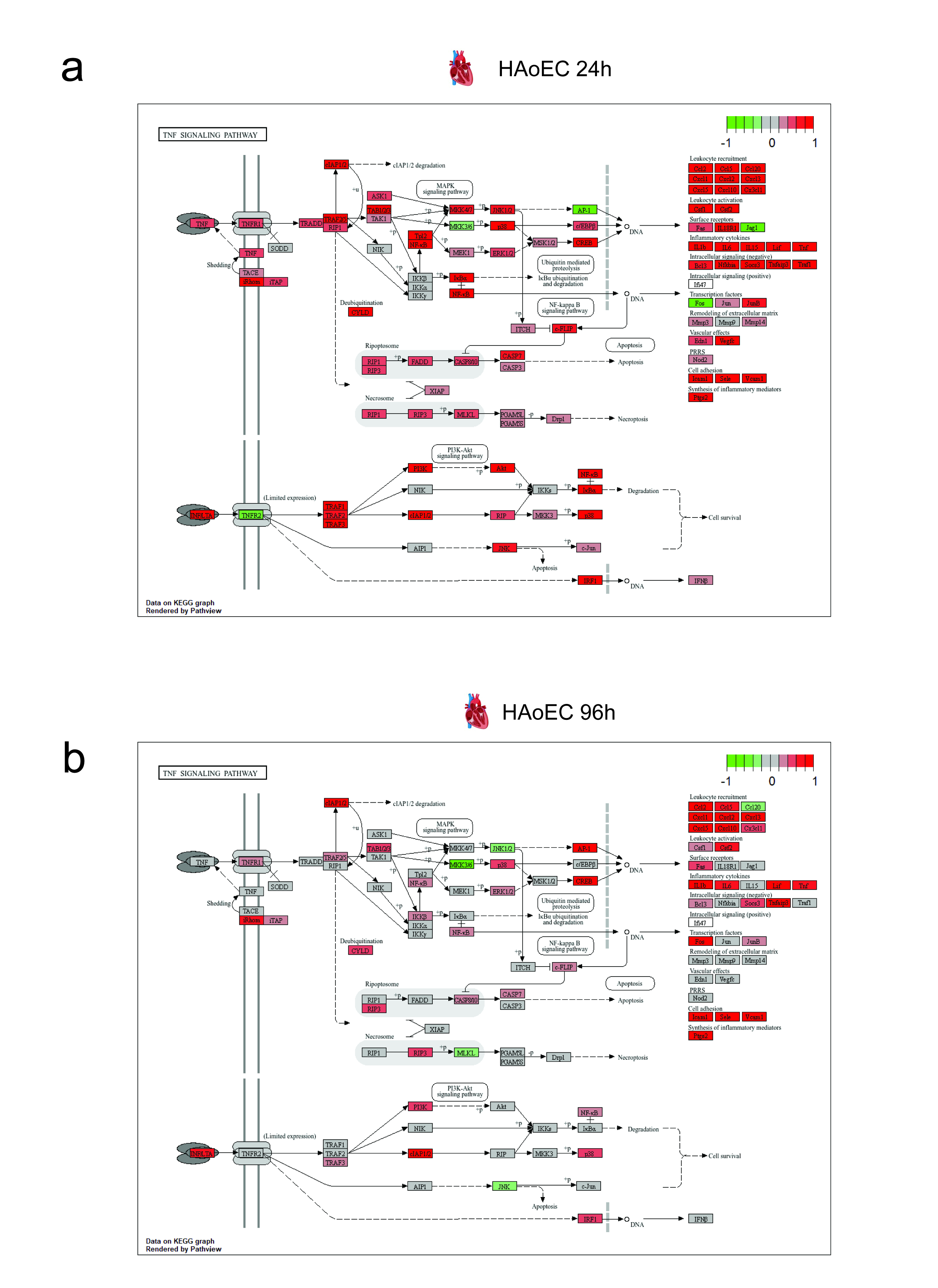

Supplement: Supplementary file 6 — Supplementary file6 A segment of the KEGG pathway showing the enrichment of the TNF signaling pathways by DE genes associated with SARS-CoV-2 spike-activated HAoEC at 24h (a) and 96h (b) (TIF 37335 KB). [file 10753_2024_2208_MOESM6_ESM.tif]

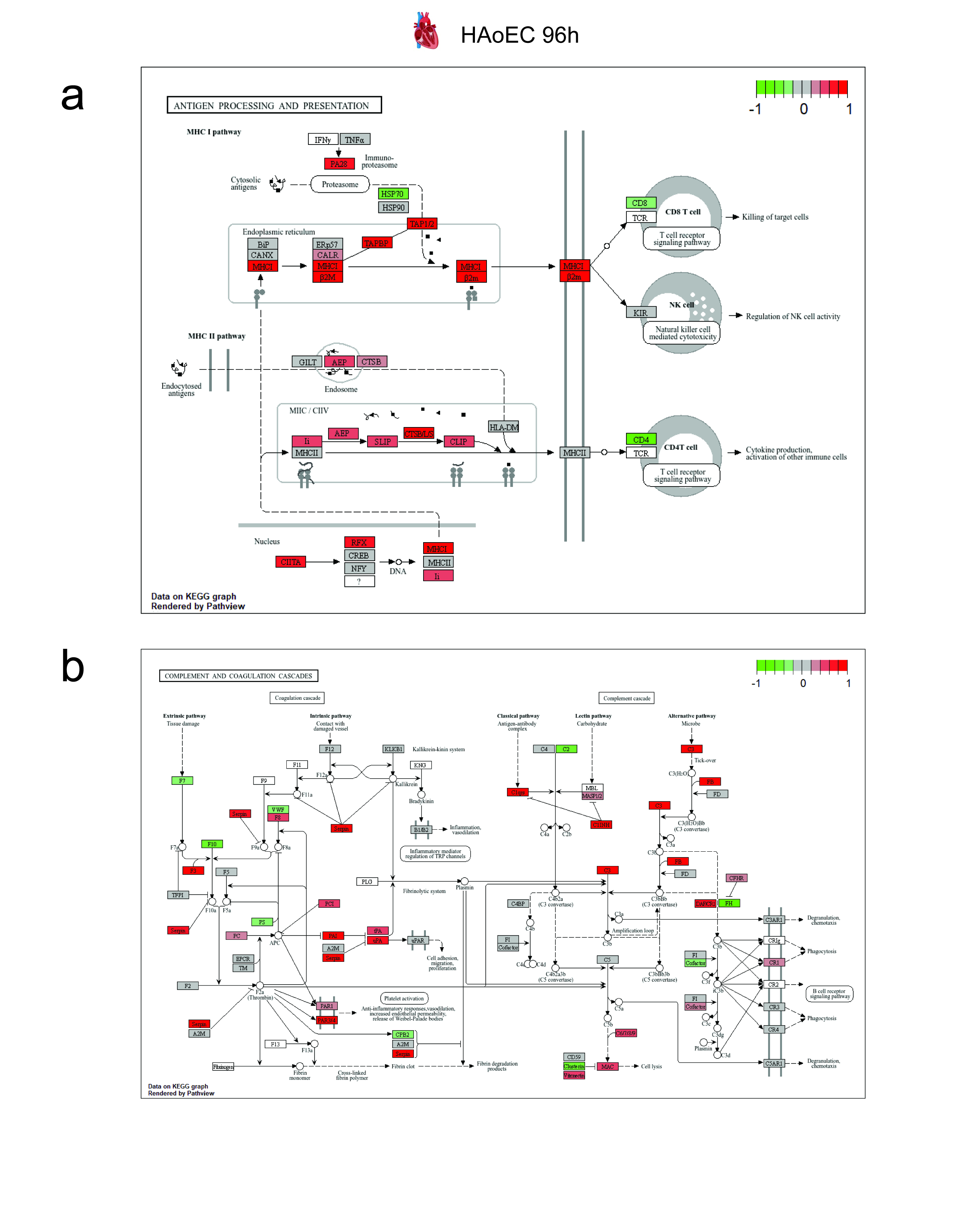

Supplement: Supplementary file 7 — Supplementary file7 Enrichment of antigen processing and presentation (a) as well as the complement and coagulation pathway (b) in the SARS-CoV-2 spike-treated HAoEC at 96h (TIF 33668 KB). [file 10753_2024_2208_MOESM7_ESM.tif]

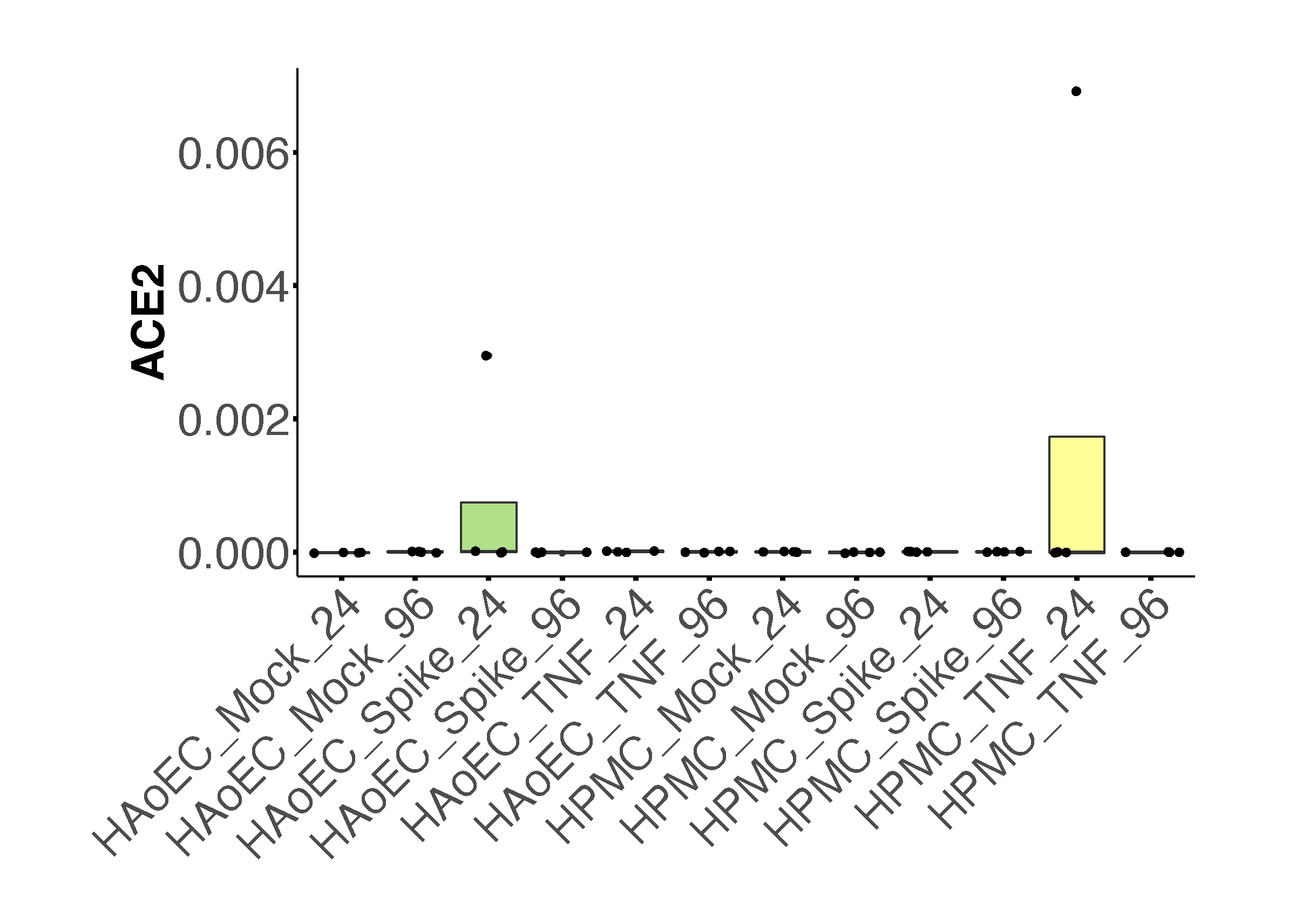

Supplement: Supplementary file 8 — Supplementary file8 Expression of ACE2 in HPMC and HAoEC. Expression level was depicted as the log-transformed of the normalized counts of each replicate (TIFF 317 KB). [file 10753_2024_2208_MOESM8_ESM.tiff]
